# Distinct cellular signatures in aplastic and intermittent phenotypes of immune effector cell-associated hematotoxicity

**DOI:** 10.1007/s00277-026-06993-3

**Published:** 2026-04-14

**Authors:** Ben Varon, Amir Grau, Noga Setter Marco, Anwar Khatib, Tsofia Levi, Riva Fineman, Inna Tzoran, Nivin Mustafa, Tsila Zuckerman, Netanel A. Horowitz, Noa Lavi, Rostislav Novak, Ofrat Beyar-Katz

**Affiliations:** 1 Department of Hematology and Bone Marrow Transplantation, Rambam Health Care Campus, 8, Ha’Aliya Street, Haifa, 3109601 Israel; 2 The Ruth and Bruce Rappaport Faculty of Medicine, Technion - Israel Institute of Technology, Haifa, Israel; 3 Biomedical Core Facility, Faculty of Medicine, Technion - Israel Institute of Technology, The Ruth & Bruce Rappaport, Haifa, Israel; 4 Cytogenetics Laboratory, Rambam Health Care Campus, Haifa, Israel; 5 Department of Orthopedic Surgery, Musculoskeletal Oncology Unit, Rambam Health Care Campus, Haifa, Israel; 6 Blood bank and Apharesis Unit, RAMBAM, Haifa, Israel; 7 Clinical Research Institute at Rambam (CRIR), Rambam Health Care Campus, Haifa, Israel

**Keywords:** ICAHT, Aplastic ICAHT, Intermittent ICAHT, CRS

## Abstract

Bone marrow toxicity, or immune effector cell-associated hematotoxicity (ICAHT), is a notable complication of CAR T-cell therapy, characterized by persistent cytopenia’s and typically classified into three phenotypes: transient, intermittent and aplastic. This study aimed to characterize the bone marrow immune landscape associated with ICAHT phenotype( aplastic and intermittent) by analyzing patients with diffuse large B-cell lymphoma (DLBCL) treated with axicabtagene ciloleucel (axi-cel) who developed ICAHT, in comparison to healthy controls and between the two ICAHT subtypes. Bone marrow samples were profiled using mass cytometry (CyTOF) with a 41-antibody panel. Compared to healthy individuals, ICAHT patients showed increased frequencies of activated/effector T cells and reduced levels of B cells and CD34^+^ early hematopoietic cells. Within the ICAHT cohort, patients with the aplastic phenotype exhibited significantly higher levels of CAR T cells, activated T cells, regulatory T cells (Tregs), Th1, and Th17 cells, along with a higher proportion of CD34^+^ early hematopoietic cells. In contrast, the intermittent phenotype was characterized by a greater abundance of naïve T cells, Th2 cells, and myeloid lineage markers such as CD33, CD11b, and CD11c. In this small, exploratory analysis, distinct immunological patterns were observed between intermittent and aplastic ICAHT phenotypes, suggesting potentially different mechanisms of hematopoietic disruption after CAR T-cell therapy that require confirmation in larger studies.

**Supplementary Information:**

The online version contains supplementary material available at 10.1007/s00277-026-06993-3.

## Introduction

Bone marrow (BM) toxicity following chimeric antigen receptor (CAR) T-cell therapy has increasingly been recognized as a significant and prevalent complication. In 2023, this toxicity was designated as immune effector cell-associated hematotoxicity (ICAHT), accompanied by a proposed grading system based on neutrophil counts [1]. Three distinct patterns of cytopenia’s have been identified post CAR T-cell therapy: transient cytopenia’s resolving quickly, recurrent cytopenia’s (intermittent phenotype), and an aplastic phenotype. The aplastic phenotype is characterized by a monophasic and prolonged period of severe neutropenia, associated with a high rate of infections and increased morbidity and mortality [2]. In contrast, the intermittent phenotype involves brief recoveries in neutrophil counts followed by recurrent episodes of neutropenia, often lasting for years, and is generally associated with a better prognosis.

The pathophysiology of ICAHT is thought to be multifactorial. In a single patient, observation suggests chronic systemic inflammation and the hyperinflammatory response during CAR T-cell migration to the bone marrow can impair the self-renewal and differentiation of hematopoietic progenitors, particularly in pre-existing hematopoietic deficits due to prior chemotherapy or senescence [3]. Moreover, the significant B-cell depletion resulting from CAR T-cell therapy disrupts the balance between T- and B-cells, promoting oligoclonal T-cell expansion [4].

Among the CD19-targeted CAR T therapies for large B-cell lymphoma, axicabtagene ciloleucel (axi-cel) is associated with a higher incidence of cytokine release syndrome (CRS), immune effector cell-associated neurotoxicity syndrome (ICANS), and hematotoxicity compared to tisagenlecleucel (tisa-cel) and lisocabtagene maraleucel (liso-cel) [5–8].

To the best of our knowledge, there is currently no data on the specific mechanisms that differentiate the intermittent and aplastic phenotypes, which could elucidate the better outcomes seen in patients with the intermittent form. This study aimed to compare the epidemiological, clinical, and laboratory characteristics of lymphoma patients treated with axi-cel exhibiting either phenotype, and to assess the bone marrow cell populations among these phenotypes and relative to those in individuals with normal bone marrow.

## Methods

### Clinical and laboratory data

CAR T-cell infusion was conducted on day 0. Prior to infusion, all participants underwent lymphodepleting chemotherapy, administered as a regimen of fludarabine and cyclophosphamide (fludarabine 25–30 mg/m^2^ from days −5 to −3, cyclophosphamide 500 mg/m^2^ from days −5to −3). CRS and ICANS were diagnosed and graded according to the American Society for Transplantation and Cellular Therapy (ASTCT) consensus grading system [9]. Grade ≥ 3 CRS was classified as severe CRS, while grade ≥ 3 ICANS was classified as severe ICANS. Early/Late ICAHT and intermittent vs aplastic phenotypes were classified based on the EHA/EBMT grading [2].

### Patients and healthy donor samples

Pre-lymphodepletion bone marrow aspirates were obtained only when deemed clinically necessary by the treating physicians, usually in cases where PET-CT suggested marrow involvement. These specimens were not stored or subjected to further analysis.

BM aspirates were collected from patients with diffuse large B-cell lymphoma (DLBCL) patients treated with axi-cel and experiencing ICAHT grade 3/4. Patients showing evidence of bone marrow lymphoma, as determined by flow cytometry, were excluded from the study.

BM mononuclear cells were isolated from BM aspirates, separated by centrifugation over a layer of Lymphoprep™ (Axis-Shield PoC AS, Norway) and then stored in freezing medium in a liquid nitrogen tank. All patients were treated with G-CSF at the time of BM aspiration. Additionally, healthy individuals undergoing orthopedic surgery due to injury were included in our study in order to obtain normal BM samples. BM cells were collected during the surgery. Mononuclear cells were processed and handled following the same protocol used for ICAHT patients. All participants provided written informed consent prior to their inclusion in the study. This study was approved by the Rambam Health Care Campus Institutional Review Board (IRB) (Helsinki Committee; approval number 0491–22-RMB) and was conducted in accordance with the Declaration of Helsinki and Good Clinical Practice guidelines.

#### Cytometry by time of flight (CyTOF)

Cryopreserved bone marrow mononuclear cells were thawed, washed, resuspended in 1mL of cell staining buffer (Standard Biotools, cat 201,068) and counted. Up to 3 million cells were used for staining. Cells were incubated with 5μL of Fc block™ (BD Biosciences, cat 564,220) and stained with the 41 metal-isotope tagged antibodies of the mass cytometry panel in a volume of 100μL on ice. Subsequently, cells were washed and incubated with 0.5mL of 1:2000 Cell-ID™ Intercalator-103Rh 500μM (Standard Biotools, cat 201103 A) for 15 min at room temperature (RT) to label dead cells. Intracellular markers were stained using the FoxP3 eBioscience set (Thermo Fisher, cat 00–5523-00) for 45 min on ice. Cells were then washed and 1mL of 1:2000 125nM of Cell ID™ Intercalator-Ir (Standard Biotools, cat 201192 A) in fix and perm buffer (Standard Biotools, cat 201,067) was added to label nucleated cells. Cells were then stored at 4 °C for up to 48 h. On the day of data acquisition, cells were washed once in staining buffer and twice in CAS solution (Standard Biotools, cat 201,244). Finally, pelleted cells were resuspended in CAS solution containing 1:10 EQ Four Element Calibration Beads (Standard Biotools, cat 201,078) at a concentration of 0.5–0.75 × 10^6^ cells/mL and acquired on the Helios™ mass cytometer (Standard Biotools). After measurement, all mass cytometry data were normalized using the EQ bead signal and the reference EQ passport P13H2302. For each acquired mass cytometry data file gaussian parameters of the Helios system were used to gate single, live CD45^+^ cells using cytobank software.

#### Antibodies

Purified antibodies were obtained from commercial vendors and labeled with metal isotopes using the MAXPAR™ X8 chelating polymer kit (Standard BioTools) following the manufacturer’s instructions.

#### Definition of immune cell populations

Major immune lineages were identified at the overview level of the tSNE analysis and were defined based on the following:

**T cells:** CD66B⁻CD19⁻CD14⁻CD11c⁻CD3⁺; **CAR T cells:** CD3⁺CAR-19⁺; **T naïve**: CD3⁺CD45RA⁺ CCR7⁺; **T central memory**: CD3⁺CD45RA⁻CCR7⁺; **T effector memory:** CD3⁺CD45RA⁻CCR7⁻; **T effector:** CD3⁺CD45RA⁺CCR7⁻; **Th1:** CD3⁺CD4⁺CXCR3⁺CCR6⁻; **Th17**:CD3⁺CD4⁺CXCR3⁻CCR6⁺;**Th2:**CD3⁺CD4⁺CXCR3⁻CCR6⁻;**Treg**:CD3⁺CD4⁺CD25⁺Foxp3⁺;**NKcells**:CD66b⁻CD19⁻CD3⁻CD14⁻HLA-DR⁻CD56⁺;**Monocytes**: CD66b⁻CD19⁻CD3⁻CD56⁻HLA-DR⁺CD11c⁺CD14⁺; **Neutrophils:** CD66b⁺HLA-DR⁻CD16⁺

#### CyTOF data analysis

tSNE-CUDA analysis was conducted using the Cytobank platform. Each run of the tSNE-CUDA algorithm was performed with the following parameters: 750 iterations, a perplexity of 30, a learning rate of 1261, and an early exaggeration factor of 12. All 52 channels were included in the analysis, excluding event, time, and width parameters. Cell populations of interest were identified from the resulting tSNE-CUDA maps and subsequently visualized and analyzed for immunophenotyping using GraphPad Prism version 10.2 for macOS.

### Flow cytometric analysis

Cryopreserved bone marrow mononuclear cells were thawed, washed, and resuspended in 1 mL of cell staining buffer. Cells were immunostained using the following antibody panel: FITC-CD38, BV421-CD34, APC/Cy7-CD45, PE-Cy7-CD45RA, PE-CD135, and PE-Cy5-CD10. All monoclonal antibodies were purchased from BD biosciences and used according to the manufacturer’s instructions.

Data were acquired on BD LSRFortessa (BD Biosciences), with a minimum of 100,000 events collected per sample. Flow cytometric data were analyzed using FlowJo software (version 10).

Hematopoietic stem cells and progenitor populations were identified within the CD45^+^ compartment based on the expression of CD34, CD38, CD45RA, CD135, and CD10. The following populations were defined:

Hematopoietic stem cells (HSC)-like (CD34⁺CD38⁻CD45RA⁻CD135⁻), multipotent progenitors (MPP; CD34⁺CD38⁻CD45RA⁻CD135⁺), common lymphoid progenitors (CLP; CD34⁺CD38⁺CD10⁺CD45RA⁺), and myeloid-biased progenitors (CD34⁺CD38⁺CD10⁻).

#### Statistical analysis

Descriptive statistics were used to summarize categorical variables (frequencies and relative frequencies) and numeric variables (median and standard deviation). Baseline characteristics were compared between groups using non-parametric tests, as appropriate. Immune cell subsets were analyzed using multiple comparisons with false discovery rate (FDR) correction according to the Benjamini–Hochberg procedure. Given the small sample size, data are presented as median (range), and individual data points are displayed in the figures. Statistical significance was defined as a two-sided *p* value < 0.05.

## Results

### Cellular signature of ICAHT compared to healthy individuals

Among 76 consecutive patients treated with axi-cel for DLBCL, 10 (13%) experienced grade 3/4 ICAHT and were in complete response at the time of bone marrow collection. Clinical characteristics of patients are described in Table 1. The median age of the patient cohort was 58.5 years (range: 39–74) with a median International Prognostic Index (IPI) of 2. Bone marrow involvement, as assessed by bone marrow biopsy, was detected in 1 of 10 patients (10%) prior to lymphodepletion. At the time of lymphodepletion, progressive disease was observed in four patients, while five patients had a partial response to bridging treatment. All patients received axi-cel as third-line therapy or beyond, with lymphodepletion using fludarabine and cyclophosphamide. None of the patients experienced hemophagocytic lymphohistiocytosis (HLH) or ith reduced blood confections associated wis.unt

**Table 1:** Patient characteristics

	All ICAHT patients (*n* = 10)	Intermittent phenotype (*n* = 5)	Aplastic phenotype (*n* = 5)
Age at time of CAR T-cell therapy (range)—years	58.5 (39–74)	51 (39–73)	65 (51–74)
Male sex—no. (%)	7 (70%)	4 (80%)	3 (60%)
Diagnosis	All DLBCL		
Transformed from indolent lymphoma	4 (40%)	2 (20%)	2 (20%)
IPI- median(range)	2(2–4)	2	2
CAR T-cell product	All treated with axi-cel		
Prior lines of therapy—median (range)	2.5 (2–3)	3 (2–3)	2 (2–3)
RCHOP	8 (80%)	4 (100%)	4(100%)
GCHOP	2 (20%)	1 (20%)	1 (20%)
ICE (with/without rituximab)	6 (60%)	2 (40%)	4 (80%)
Gemcitabine/GemOx	4 (40%)	3 (60%)	1 (20%)
Other	4 (40%)	3 (60%)	1 (20%)
Disease status prior to CAR T-cell infusion			
CR	1 (10%)	0	1 (20%)
PR	5 (50%)	3 (60%)	2 (40%)
PD	4 (40%)	2 (40%)	2 (40%)
BM involvement by PET-CT prior to CAR T infusion	2 (20%)	1 (20%)	1 (20%)
BM involvement by BMB prior to CAR T infusion	1 (10%)	1 (20%)	0
Lymphodepletion therapy	All received fludarabine + cyclophosphamide		
CAR-HEMATOTOX score—median (range)	0.5	0 (0–1)	2 (0–4)
CRS	10 (100%)	5 (100%)	5 (100%)
Grade 1–2	7 (70%)	4 (80%)	3 (60%)
Grade 3–4	3 (30%)	1 (20%)	2 (40%)
ICANS	5 (50%)	2 (20%)	3 (60%)
Grade 1–2	3 (30%)	1 (20%)	2 (40%)
Grade 3–4	1 (10%)	1 (20%)	0
Grade 5	1 (10%)	0	1 (20%)
ICAHT grade*			
Early grade 1–2	7 (70%)	4 (80%)	3 (60%)
Early grade 3–4	3 (30%)	1 (20%)	2 (40%)
Late grade 1–2	0	0	0
Late grade 3–4	10 (100%)	5	5
G-CSF response—yes (%)	6 (60%)	5 (100%)	1 (20%)
Thrombocytopenia—yes (%)	8 80%)	3 (60%)	5 (100%)
Number of days from CAR T infusion to ealy ICAHT—median (range)	2 (1–11)	2 (2–10)	2 (1–11)
Number of days from CAR T infusion to BMB—median (range)	36 (21–573)	80 (35–573)	29 (21–37)
Bone marrow OGM analysis performed—yes (%)	5 (50%)	3 (60%)	2 (40%)
Normal	4 (40%)	2 (40%)	2 (40%)
Absence of Y chromosome	1 (10%)	1 (20%)	0
Infection(s)—yes(%)			
Bacteremia/sepsis	5 (50%)	0	5 (100%)
Chronic sinusitis	2 (20%)	2 (40%)	0
Viral respiratory tract infection	2 (20%)	1 (20%)	1 (20%)
Other	3 (30%)	1 (20%)	2 (40%)
None	2 (20%)	2 (40%)	0
Treatment for ICAHT			
Filgastrim	10 (100%)	5 (100%)	5 (100%)
Eltrombopag	3 (30%)	0	3 (60%)
Outcome—alive**	8 (80%)	5 (100%)	3 (60%)

Abbreviations: *Axi-cel* Axicabtagene ciloleucel, *BMB* bone marrow biopsy, *CAR* Chimeric antigen receptor, *CR* complete response, *CRS* cytokine release syndrome, *DLBCL* Diffuse large B-cell lymphoma, *FL* follicular lymphoma, *ICAHT* Immune effector cell–associated hematotoxicity, *ICANS* Immune effector cell-associated neurotoxicity syndrome (ICANS), *IVIG* intravenous immunoglobulins, *IPI* International Prognostic Index, *MZL* marginal zone lymphoma, *OGM* optical genome mapping, *PR* partial response, *RSV URTI* Respiratory syncytial virus Upper respiratory tract infection

*One patient died before reaching the timepoint definition of late ICAHT

** One patient died from sepsis, and the other from cerebral herniation

**Fig. 1:**
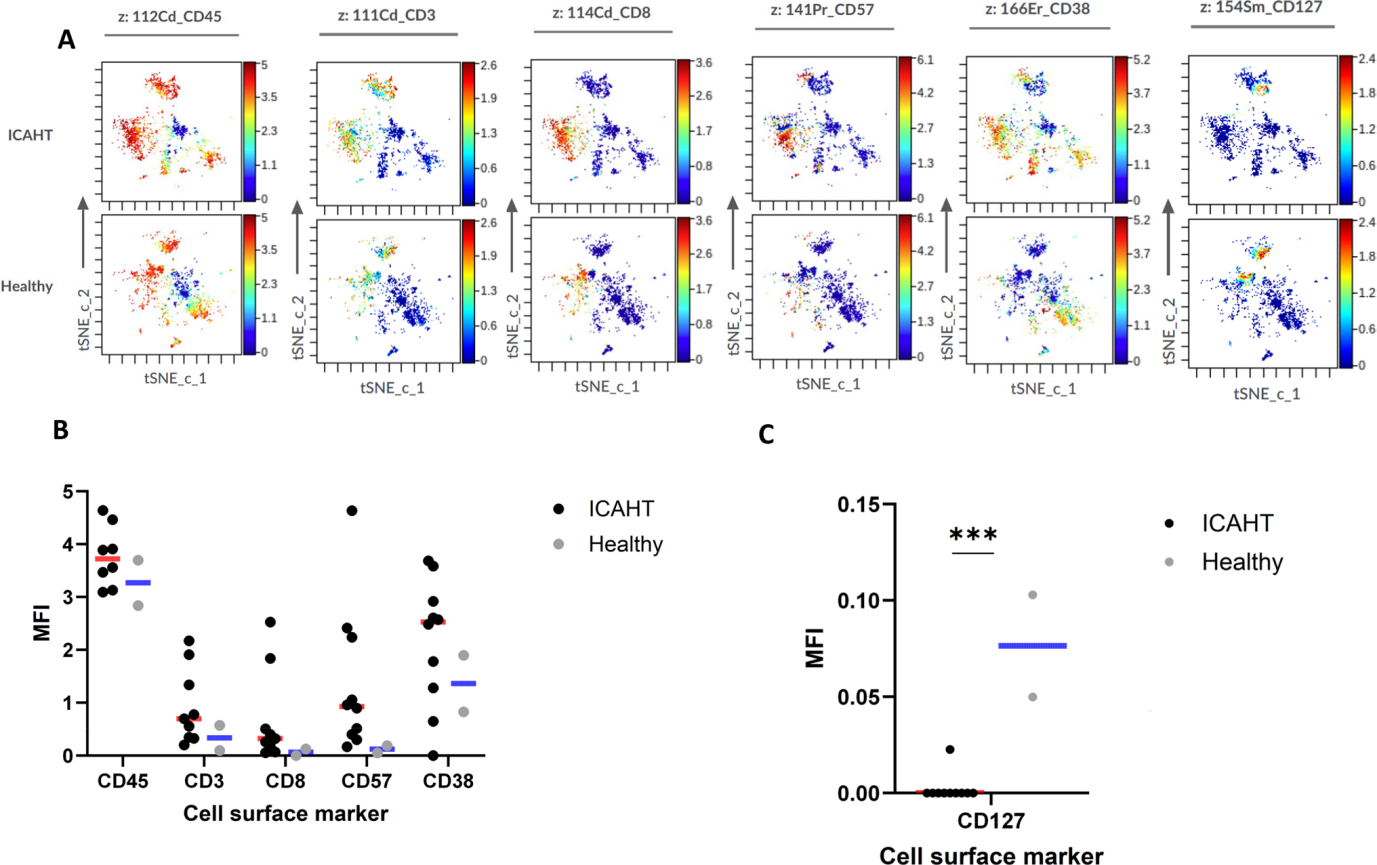
Altered Immune Marker Expression in ICAHT Patients Compared to Healthy Controls. Expression levels of immune markers were assessed using t-SNE visualization and median fluorescence intensity (MFI) analysis in bone marrow (BM) samples from ICAHT patients and healthy controls. **A** t-SNE analysis illustrating distinct immune cell clustering and marker expression patterns between ICAHT patients and healthy individuals. **B** MFI comparison of immune markers upregulated in ICAHT patients. **C** MFI comparison of markers found to be downregulated in ICAHT patients. *n* = 10 ICAHT patients; *n* = 2 healthy controls

We first compared the cellular signatures of all ICAHT patients to those of two healthy individuals undergoing orthopedic surgery due to accidental trauma, with no known bone marrow-related disorders. Quantitatively, ICAHT patients exhibited higher expression levels of CD45, CD3, CD8, CD57, and CD38 compared to healthy controls (Fig. 1A and B). In contrast, CD127 expression levels were notably higher in the healthy individuals (Fig. 1C).

**Fig. 2:**
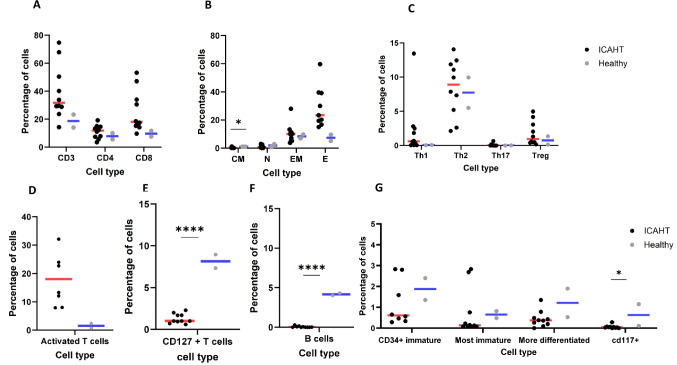
Immune Cell Signatures in ICAHT Patients Compared to Healthy Controls. Immune cell populations were analyzed in bone marrow samples using t-SNE visualization and manual gating strategies. **A** Distribution of total T cells in ICAHT patients and healthy individuals. **B** Frequencies of T cell subsets between ICAHT patients and healthy individuals. **C** Comparative analysis of T helper (Th) and regulatory T (Treg) cell populations in ICAHT patients and healthy individuals. **D** Proportions of activated T cells in ICAHT patients and healthy individuals. **E** Frequency of CD127-expressing T cells in ICAHT patients and healthy individuals. **F** Distribution of B cells in ICAHT patients and healthy individuals. **G** CD34^+^ stem cell populations in ICAHT patients compared to healthy individuals. *n* = 10 ICAHT patients; *n* = 2 healthy controls

T cells were more abundant in ICAHT patients than in healthy individuals, though the difference was not statistically significant (median: 31% vs. 18%, *p* = 0.18) (Fig. 2A). Among these, CD8^+^ T cells constituted a higher proportion of total T cells in ICAHT patients compared to healthy controls (median: 18% vs 9%, *p* = 0.17) (Fig. 2A). Analysis of T cell subtypes revealed that ICAHT patients had a higher percentage of effector T cells (23% vs. 7%, *p* = 0.07) and a significantly lower percentage of central memory T cells (0.2% vs. 1%, *p* = 0.01) (Fig. 2B). No significant differences were observed in T helper (including Th1, Th2 or Th12 subtypes) or T regulatory cell populations (Fig. 2C).

Interestingly, activated T cells, defined by co-expression of CD38 and HLA-DR, showed a trend toward an increase in ICAHT patients compared to healthy controls (18% vs. 1.5%, *p* = 0.09) (Fig. 2D). Additionally, CD127-expressing T cells were significantly reduced in the bone marrow of ICAHT patients compared to healthy controls (1% vs. 8.1%, *p* = 0.00001) (Fig. 2E).

B cells, identified by CD19^+^ and CD20^+^ markers, were significantly reduced in ICAHT patients relative to healthy individuals (0.013% vs. 4.1%, *p* = 0.000001) (Fig. 2F). We observed alterations in phenotypically immature CD34^+^ compartments in ICAHT patients compared with healthy controls; however, these differences did not reach statistical significance. Specifically, ICAHT patients exhibited reduced frequencies of both the most immature progenitor cells (CD34^+^CD38^−^) and more differentiated CD34^+^ (CD34^+^CD38^+^) subsets (Fig. 2G). In contrast, CD117^+^ cells were significantly more abundant in healthy controls than in ICAHT patients (0.6% vs. 0.04%, *p* = 0.01; Fig. 2G). Complementary flow cytometry analysis suggested a reduced HSC-like CD34⁺CD38⁻CD45RA⁻CD135⁻population in ICAHT patients compared with a healthy control, while MPP, CLP, and myeloid progenitor populations appeared similar (Supplementary Fig. 1).

### T cell immune signature of aplastic phenotype vs intermittent

The median age of patients in the intermittent group was 51 years (range: 39–73), while in the aplastic group it was 65 years (range: 51–74) (Table 1). The majority of patients in both groups were male. Patients who developed an aplastic phenotype had a median CAR-HEMATOTOX score of 2 as compared with 0 in the intermittent phenotype. All patients experienced cytokine release syndrome (CRS), with high-grade CRS occurring in 20% of the intermittent group compared to 40% in the aplastic group. Bacterial infections were observed in all patients with the aplastic phenotype but in none of the patients with the intermittent phenotype. At the time of analysis, all patients in the intermittent group were alive, whereas 60% of patients in the aplastic group remained alive.

**Fig. 3:**
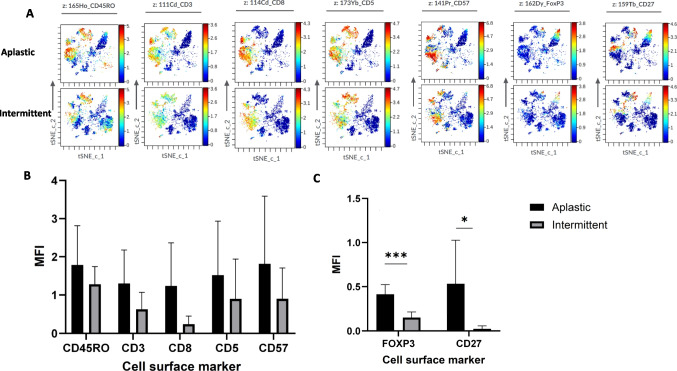
Altered Immune Marker Expression in Aplastic vs. Intermittent ICAHT Patients. Expression levels of immune markers were analyzed in BM samples from ICAHT patients using t-SNE visualization and MFI analysis. **A** t-SNE plots showing distinct immune cell clustering and marker expression patterns between aplastic and intermittent ICAHT patient subgroups. **B** MFI comparison of immune markers that did not show statistically significant differences between the two subgroups. **C** MFI comparison of immune markers that were significantly altered between aplastic and intermittent ICAHT patients. *n* = 5 aplastic ICAHT patients; *n* = 5 intermittent ICAHT patients

The median interval from CAR T infusion to bone marrow CyTOF analysis was 29 days for patients with the aplastic phenotype and 80 days for those with the intermittent phenotype. We first reviewed the cellular signatures of aplastic patients to those with intermittent phenotype. Numerically, aplastic patients exhibited higher expression levels of CD45RO, CD3, CD8, CD5, CD57, with a significant increase in CD27 and FOXP3 compared to intermittent phenotype (Fig. 3).

**Fig. 4:**
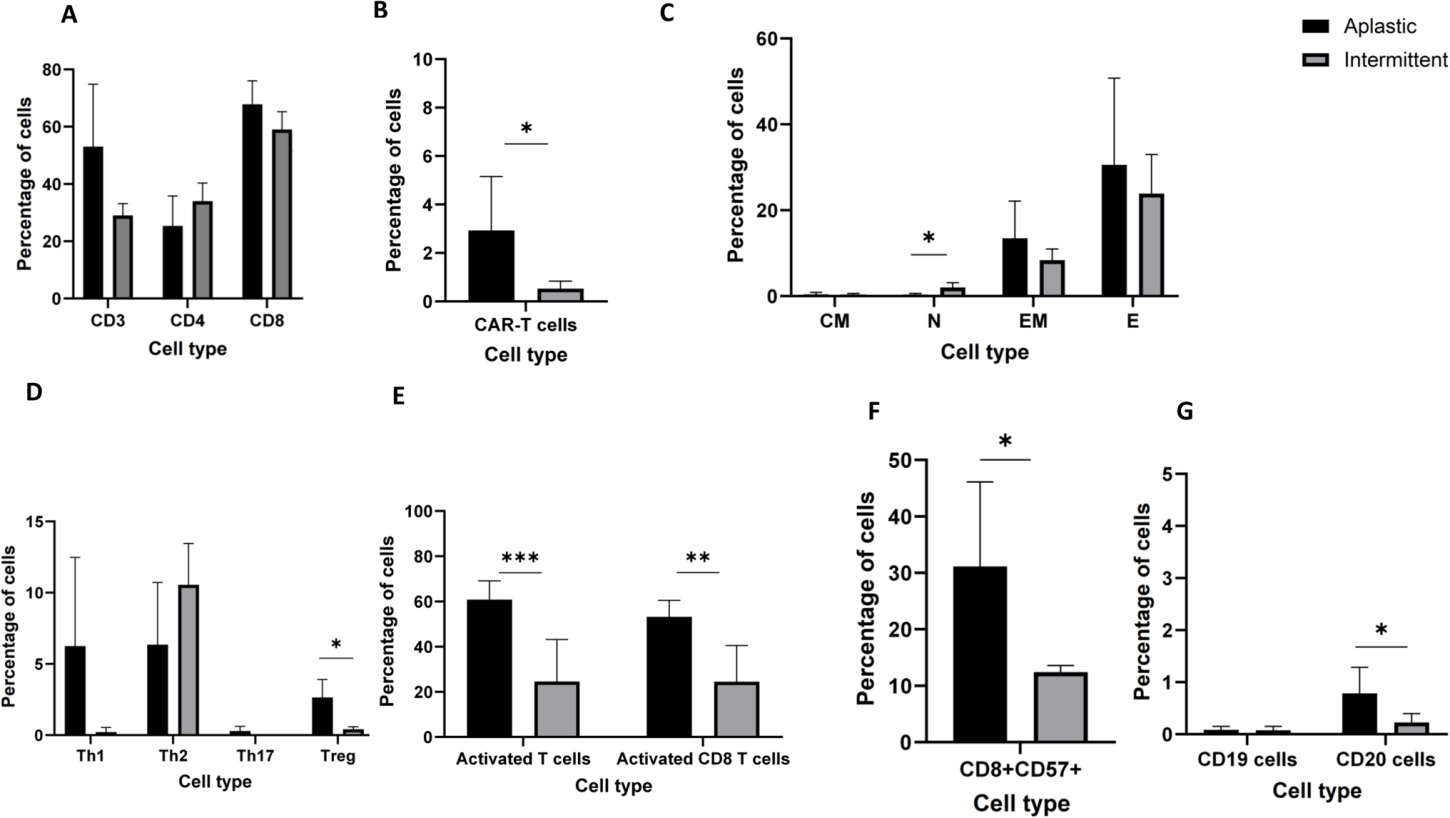
Lymphocyte Cell Signatures in Aplastic vs. Intermittent ICAHT Patients. Immune cell populations in bone marrow samples from ICAHT patients were analyzed using t-SNE visualization and manual gating strategies. **A** Distribution of total T cells in aplastic versus intermittent ICAHT patients. **B** Frequencies of CAR T cells in aplastic versus intermittent ICAHT patients. **C** Comparative analysis of T cell subset distributions between aplastic and intermittent patients. **D** Frequencies of T helper (Th) and regulatory T (Treg) cells in aplastic versus intermittent ICAHT patients. **E** Proportions of activated T cells in aplastic versus intermittent patients. **F** Frequency of CD57-expressing CD8⁺ T cells across patient groups. **G** Distribution of B cells in aplastic versus intermittent ICAHT patients. *n* = 5 aplastic ICAHT patients; *n* = 5 intermittent ICAHT patients

We next compared the frequencies of T cell subsets and CAR T cells between the intermittent and aplastic phenotypes. T cells were more abundant in the BM of patients with the aplastic phenotype compared to those with the intermittent phenotype (54% vs 29%, *p* = 0.07) with no significant change between CD8 and CD4 subtypes (Fig. 4A). CAR T-cell levels were higher in the aplastic patients (2.7% vs. 0.56%, *p* = 0.04) (Fig. 4B). The only T-cell subtype showing a notable difference was naïve T-cells, which were higher in the intermittent patients (2.3% vs. 0.3%, *p* = 0.02) (Fig. 4C). The Th1 and Th17 phenotypes, known for their roles in regulating pro-inflammatory responses [10], were more prevalent in the aplastic patients, while Th2 cells were observed more frequently in the intermittent phenotype (Fig. 4D). T regulatory cells were significantly higher in the aplastic phenotype compared to intermittent (2.6% vs 0.4%, *p* = 0.01) (Fig. 4D). Notably, the percentages of CD38^+^ and HLA-DR^+^ T cells, as well as CD8^+^ CD38^+^ HLA-DR^+^ T cells (considered as activated T-cells [11, 12](, were significantly higher in the aplastic phenotype (61% vs. 20%, *p* = 0.004; 55% vs. 19%, *p* = 0.006) (Fig. 4E). Furthermore, the proportion of CD8⁺CD57⁺ T cells was significantly higher in aplastic patients compared to intermittent phenotype (31% vs. 12%, *p* = 0.046) (Fig. 4F). When comparing CAR-positive cells with non-CAR-expressing T cells, activation marker expression was significantly higher in both populations in the aplastic phenotype compared with the intermittent phenotype (Supplementary Fig. R-T2A). Within the CAR-expressing T-cell compartment, the intermittent group exhibited a higher proportion of naïve cells. Similarly, among CAR-negative T cells, the intermittent group also showed an increased frequency of naïve cells, whereas the aplastic group was enriched for EM T cells (Supplementary Fig. 2B–C).

As expected following CD19-directed CAR T-cell therapy, all patients with ICAHT exhibited low levels of CD19^+^ cells in the bone marrow, with no significant differences observed between those with the aplastic and intermittent phenotypes(Fig. 4 G). However, a significant reduction in the percentage of CD20^+^ cells was observed in patients with the intermittent phenotype compared to those with the aplastic phenotype (0.19% vs. 0.76%, *p* = 0.04) (Fig. 4G).

### Stem cells progenitors and innate immune cell signature of aplastic phenotype vs intermittent

**Fig. 5:**
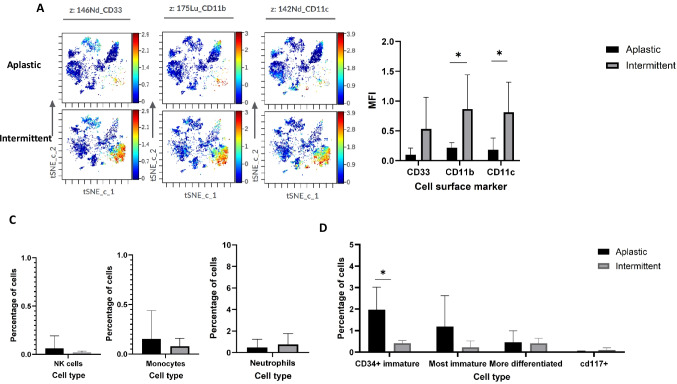
Myeloid Cells, NK Cells, and Stem Cell Signatures in Aplastic vs. Intermittent ICAHT Patients. Expression levels of immune markers were analyzed in BM samples from ICAHT patients using t-SNE visualization and MFI analysis. **A** t-SNE plots illustrating differences in immune cell clustering and marker expression patterns between aplastic and intermittent ICAHT patient subgroups. **B** MFI comparison of selected immune markers between the two subgroups. **C** Frequencies of innate immune cell populations, including natural killer (NK) cells, monocytes and neutrophils in aplastic versus intermittent patients. **D** Comparison of CD34^+^ cell populations between the two groups. *n* = 5 aplastic ICAHT patients; *n* = 5 intermittent ICAHT patients

We first reviewed the cellular signatures of aplastic patients to those with intermittent phenotype. The intermittent group showed an increase in CD33, CD11b, and CD11c expression, consistent with enrichment of cells exhibiting a myeloid-associated immunophenotype (Fig. 5A and B). No significant differences were found in the frequencies of natural killer cells, monocytes and neutrophils (Fig. 5C). When comparing the aplastic and intermittent phenotypes, we observed a significantly higher proportion of CD34^+^ cells in the aplastic group (2.1% vs. 0.4%, *p* = 0.02; Fig. 5D).

## Discussion

In this study, we performed immune profiling of bone marrow samples from patients with ICAHT following axi-cel therapy, focusing specifically on two clinically distinct phenotypes: aplastic and intermittent. These analyses are exploratory and aimed at identifying numerical trends rather than definitive statistical differences, given the small sample size. Our data suggest potential immunological and hematopoietic differences between ICAHT patients and healthy controls, as well as between the aplastic and intermittent phenotypes. Compared to healthy individuals, ICAHT patients exhibited a trend towards expansion of CD8⁺ T cells, activated T cells (CD38⁺HLA-DR⁺), and effector T cell subsets, accompanied by reductions in B cells and early hematopoietic progenitors. Within the ICAHT cohort, the aplastic phenotype was associated with higher frequencies of CAR T cells, activated T cells, and CD8⁺CD57⁺ T cells, as well as a notable increase in T regulatory cells. In contrast, the intermittent phenotype displayed a more diverse T-cell profile, including higher proportions of naïve and Th2 T cells, and greater representation of myeloid lineage markers such as CD33, CD11b, and CD11c. These observations should be interpreted cautiously and require validation in larger patient cohorts. The reduction of CD127-expressing T cells in the bone marrow of ICAHT patients compared to healthy controls may reflect immune dysregulation and T cell exhaustion commonly observed in chronic inflammatory. CD127, the α-chain of the IL-7 receptor, is crucial for T cell survival, homeostasis, and memory formation. Decreased CD127 expression is often associated with T cell activation and exhaustion, a state characterized by impaired effector function and proliferative capacity [13]. This decrease may contribute to impaired immune reconstitution and tolerance breakdown observed in ICAHT patients.

Numerous studies have emphasized the critical role of activated T-cells in a variety of immune syndromes, illustrating their involvement in both the initiation and perpetuation of inflammatory responses. These activated T-cells, particularly those expressing markers such as CD38 and HLA-DR, are implicated in diverse conditions ranging from autoimmune disorders to infections, indicating their importance in immune regulation and pathogenesis. For instance, CD8^+^ HLA-DR^+^ T cells may contribute to severe bone marrow failure in aplastic anemia [14]. Additionally, HLA-DR^+^ CD38hi T cells have been identified across a spectrum of hyperinflammatory and immune dysregulation disorders, showing a correlation with soluble IL-2 levels [15]. In patients with severe COVID-19, a persistent accumulation of HLA-DR^+^ CD38hi CD8^+^ T cells has been linked to systemic inflammation, tissue injury, and immune dysfunction, further underscoring the significance of these activated T cells in inflammatory responses [16].

The higher abundance of activated T cells and terminally differentiated CD8⁺CD57⁺ T cells in the aplastic phenotype suggests a more sustained and possibly deleterious T-cell activation state within the BM microenvironment. CD8⁺CD57⁺ T cells have been associated with senescence and cytotoxicity, and their accumulation could contribute to the suppression of hematopoietic progenitors and the prolonged neutropenia seen in this group [17–19].

Notably, CAR T-cell persistence was higher in patients with the aplastic phenotype. While long-term CAR T-cell persistence is generally associated with durable anti-tumor responses [20], it may also lead to prolonged immune effector activity and collateral damage to the BM niche. The elevated Th1 and Th17 profiles in the aplastic group further implicate pro-inflammatory cytokine signaling as a contributor to BM toxicity, potentially exacerbating progenitor cell exhaustion or apoptosis [10, 21–23].

Conversely, patients with the intermittent phenotype exhibited signs of partial immune reconstitution, including higher proportions of naïve T cells and a trend toward increased myeloid lineage cells. These findings suggest a more balanced immune environment that may allow for episodic recovery of hematopoiesis, consistent with the fluctuating neutrophil counts observed clinically. The predominance of Th2 cells in this group may indicate an anti-inflammatory tendency that favors hematopoietic recovery, or at least mitigates the suppressive effects of chronic inflammation [10, 24].

Interestingly, while CD19^+^ B cells were absent in all patients, residual CD20⁺ cells were more prevalent in the aplastic group. This may reflect differences in B-cell maturation arrest or selective depletion patterns post-CAR T therapy and warrants further investigation.

HSCs possess the unique capability to differentiate into all types of mature blood cells, including red blood cells, white blood cells, and platelets. This process of differentiation is intricately regulated by a variety of factors, including the cellular microenvironment, signaling molecules, and interactions with surrounding immune cells [25]. The higher CD34⁺ immature cells in aplastic versus intermittent patients may indicate impaired progenitor differentiation, warranting further investigation in larger studies.

A recent study investigating cytopenia’s in multiple myeloma patients treated with anti-BCMA CAR T cells demonstrated that, in an ex vivo model, supernatants from activated CAR T cells inhibited the differentiation of hematopoietic stem and progenitor cells (HSPCs), resulting in a more immature cellular phenotype. These results indicate a potential paracrine effect of CAR T cells that may contribute to HSPC maturation arrest [26].

Our study is limited by the small sample size, particularly within the intermittent and aplastic subgroups, which may limit the statistical power and generalizability of our findings. Additionally, the cross-sectional nature of the sampling precludes temporal analysis of immune reconstitution or the evolution of BM signatures over time. Longitudinal studies with serial BM sampling, combined with cytokine profiling and single-cell transcriptomics, will be crucial to fully delineate the mechanisms driving ICAHT phenotypes. These insights may inform therapeutic interventions such as immunomodulation or stem cell support in patients with persistent cytopenia’s. Finally, the technique used in this study did not obtain information regarding the BM stroma and BM granulocytes which may have a substantial effect on efficacy and toxicity.

## Conclusion

Our findings suggest distinct immunophenotypic and hematopoietic patterns in aplastic and intermittent ICAHT phenotypes following CD19 CAR T-cell therapy compared with normal bone marrow. The aplastic phenotype was associated with higher frequencies of activated T cells, an inflammatory profile, and increased CD34⁺ cells, whereas the intermittent phenotype showed signs of partial immune reconstitution. Although based on a small cohort, these observations may provide preliminary insight into CAR T-cell-associated bone marrow changes and could help guide future studies aimed at understanding risk factors and potential interventions.

## Supplementary Information

Below is the link to the electronic supplementary material.

Supplementary file1 Supplementary Figure 1: Hematopoietic stem cell population by flow cytometry. Immune cell populations in bone marrow samples from ICAHT patients were analyzed using flow cytometry. *n* = 5 ICAHT patients; *n* = 1 healthy controls. (PPTX 153 KB)

Supplementary file2 Supplementary Figure 2: CAR-T-positive versus CAR-T-negative T-cell populations. Immune cell populations in bone marrow samples from ICAHT patients were analyzed using t-SNE visualization and manual gating strategies. (A) Proportions of activated CAR-T-positive cells and CAR-negative T cells in patients with aplastic versus intermittent phenotypes. (B) Comparison of cell subset distributions between aplastic and intermittent phenotypes within the CAR-T-positive compartment. (C) Comparison of cell subset distributions between aplastic and intermittent phenotypes within the CAR-negative T-cell compartment. *n* = 5 aplastic ICAHT patients; *n* = 5 intermittent ICAHT patients. (PPTX 598 KB)

## Data Availability

The data that support the findings of this study are available from the corresponding author upon reasonable request. The data are not publicly available due to ethical and privacy restrictions related to patient information.
