# Supplementary figures and images for: Distinct cellular signatures in aplastic and intermittent phenotypes of immune effector cell-associated hematotoxicity
Source: Ann Hematol. 2026 Apr 14;105(5):243. doi: 10.1007/s00277-026-06993-3 (PMC13079477; doi:10.1007/s00277-026-06993-3)

## Slide 1
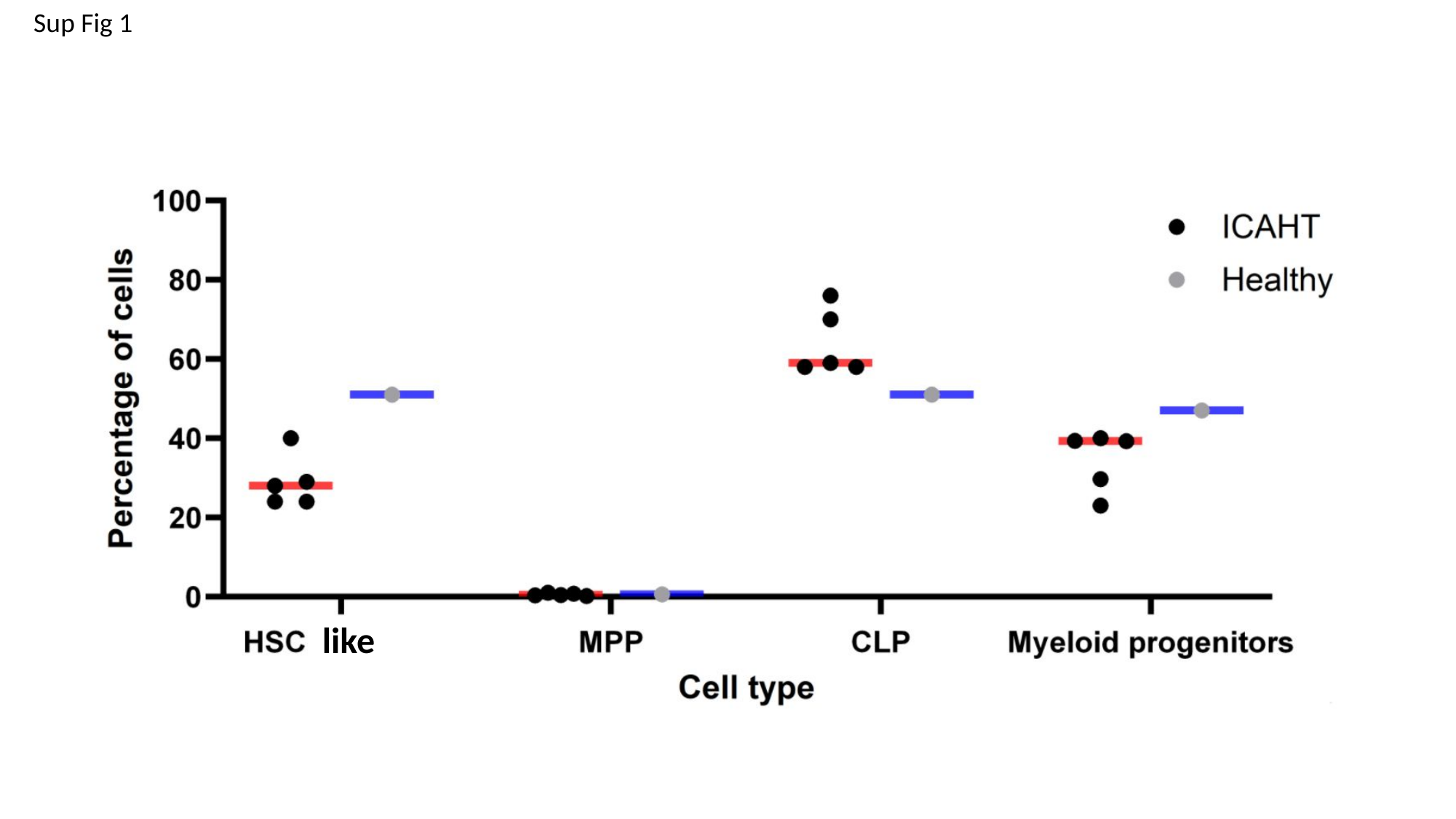

Sup Fig 1
like

Supplement: Supplementary file 1 — Supplementary file1 Supplementary Figure 1: Hematopoietic stem cell population by flow cytometry. Immune cell populations in bone marrow samples from ICAHT patients were analyzed using flow cytometry. n = 5 ICAHT patients; n = 1 healthy controls. (PPTX 153 KB) [file 277_2026_6993_MOESM1_ESM.pptx]

## Slide 1
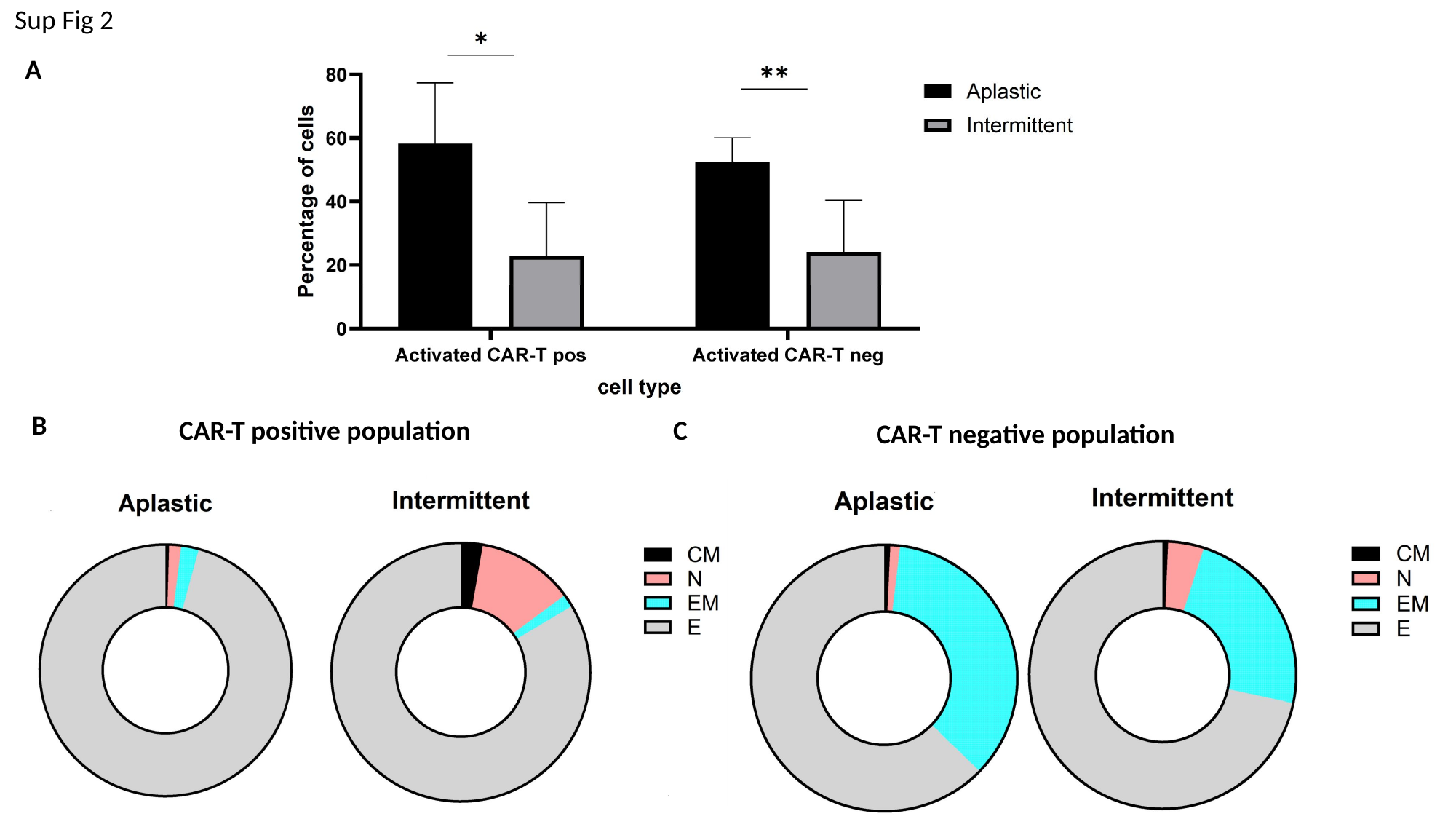

Sup Fig 2
A
B
CAR-T positive population
C
CAR-T negative population

Supplement: Supplementary file 2 — Supplementary file2 Supplementary Figure 2: CAR-T-positive versus CAR-T-negative T-cell populations. Immune cell populations in bone marrow samples from ICAHT patients were analyzed using t-SNE visualization and manual gating strategies. (A) Proportions of activated CAR-T-positive cells and CAR-negative T cells in patients with aplastic versus intermittent phenotypes. (B) Comparison of cell subset distributions between aplastic and intermittent phenotypes within the CAR-T-positive compartment. (C) Comparison of cell subset distributions between aplastic and intermittent phenotypes within the CAR-negative T-cell compartment. n = 5 aplastic ICAHT patients; n = 5 intermittent ICAHT patients. (PPTX 598 KB) [file 277_2026_6993_MOESM2_ESM.pptx]
